# Perceptions of cancer survivors and healthcare professionals on adequate supportive care after treatment of cancer: An in-depth qualitative study

**DOI:** 10.1371/journal.pone.0355113

**Published:** 2026-08-25

**Authors:** Doris van der Smissen, Marjolein Lugtenberg, Yosra Abdeselam Rocdi, Laurens V. Beerepoot, Floortje Mols, Evelien P. M. Brouwers, Dareczka K. Wasowicz, Margot C. W. Joosen

**Affiliations:** 1 Tranzo, Scientific Center for Care and Wellbeing, Tilburg School of Social and Behavioral Sciences, Tilburg University, Tilburg, The Netherlands; 2 Department of Dermatology, Erasmus MC Cancer Institute, University Medical Center Rotterdam, Rotterdam, The Netherlands; 3 Department of Internal Medicine, Elisabeth-TweeSteden Hospital, Tilburg, The Netherlands; 4 Department of internal oncology, Erasmus MC Cancer Institute, University Medical Center Rotterdam, Rotterdam, The Netherlands; 5 Center of Research on Psychological disorders and Somatic diseases, Department of Medical and Clinical Psychology, Tilburg University, Tilburg, The Netherlands; 6 Department of Surgery, Elisabeth-TweeSteden Hospital, Tilburg, The Netherlands; Universiti Sains Malaysia - Kampus Kesihatan, MALAYSIA

## Abstract

**Objective:**

Following treatment, cancer survivors often struggle to resume their life in several life domains. Although supportive care is increasingly available and can assist survivors in improving their quality of life, it often falls short in meeting the needs of the diverse and growing group of cancer survivors. Understanding the perceptions of key stakeholders is crucial for improving supportive care. This study aims to explore the perceptions of cancer survivors and healthcare professionals (HCPs) regarding the provision of adequate supportive care after treatment.

**Methods:**

We conducted 22 interviews with cancer survivors, varying in (time since) diagnosis, sex and age. Additionally, four focus groups were conducted with 25 HCPs, including medical specialists, general practitioners, psychologists, occupational physicians and informal caregivers. Interviews and focus groups were conducted using pre-defined topic guides, were audio-taped, and transcribed verbatim. A thematic content analysis was performed, consisting of several coding phases.

**Results:**

Cancer survivors emphasized the importance of a person-centered approach, with supportive care tailored to individual values and needs. They valued accessible supportive care extending beyond medical needs, including work-related support and peer support. HCPs similarly stressed the importance of person-centered supportive care, and promoting cancer survivors’ empowerment. They also highlighted limited awareness of supportive care options among HCPs, the need for clear role division, multidisciplinary collaboration, integration into clinical care, and an overview of supportive care referral options.

**Conclusion:**

While both cancer survivors and HCPs emphasized the importance of comprehensive, person-centered supportive care, its implementation in clinical practice remains challenging. Key requirements, such as greater awareness of the importance of supportive care among HCPs, knowledge of reliable referral options, and interdisciplinary collaboration, are often unmet. Efforts should focus on improving HCPs’ awareness and integrating supportive care into clinical practice. The findings of this study informed the development of the online tool ‘Back on Track with Cancer’. This tool supports the identification of supportive care needs, provides personalized supportive care options, and facilitates shared decision making with HCPs.

## Introduction

Advancements in cancer treatment and cancer screening have significantly increased the number of cancer survivors over recent decades [1,2]. Studies underscore that survivors of various types of cancer may experience several long-term effects of disease and treatment, including physical complaints, anxiety, fatigue and reduced quality of life [2–8]. Moreover, primarily qualitative studies indicate that cancer survivors often face various challenges in resuming life in several life domains, related to work, finances, relationships and social life [2,7,9–14]. This impact is observed, not only among advanced cancer survivors [9–11,15], but also among those with lower-stage cancers [14, 16, 17].

While previous research has mainly focused on specific cancer survivor groups [2–6], a recently conducted qualitative study explored the overarching experiences of cancer survivors in resuming life after treatment, transcending the type of cancer diagnosis [14]. This study revealed that cancer survivors face numerous challenges that extend beyond a specific diagnosis and treatment [14]. Following treatment, survivors may experience a (temporary) fall-out episode and often struggle to resume their life in several life domains [14]. In addition, cancer survivors have to cope with all the changes associated with the disease and its treatment and have to regain confidence in themselves [14]. This process often leads to a re-evaluation of values in life, health and work [14]. These overarching experiences should be taken into account by using a transdiagnostic approach in supportive care, by focusing on the exploration of underlying needs and interventions transcending individual diagnoses [18,19] when developing interventions to help survivors to resume their lives.

Providing adequate supportive care can support cancer survivors to deal with the challenges associated with cancer and the treatment. Supportive care can support participation and improve quality of life of cancer survivors, while it may reduce medical costs by timely and low-threshold support also outside of the expensive medical hospital care [20]. Supportive care is defined as patient-centered care across the disease trajectory, from diagnosis to survivorship and including end-of-life care, encompassing physical, psychological, social and spiritual well-being to improve quality of life [21]. This encompasses not only supportive care inside the hospital, but also outside of the hospital including informal care [21]. While existing supportive care research – particularly qualitative studies – predominantly focuses on specific cancer populations [10, 22], few studies have explored the overarching needs of cancer survivors [23, 24]. Consequently, a comprehensive understanding of survivors’ perceptions regarding adequate supportive care is lacking. Although cancer survivors experience physical, psychological and psychosocial needs for supportive care, current supportive care following cancer treatment often falls short in meeting the needs of the diverse and growing group of cancer survivors [23,25–27].

Understanding the perceptions of key stakeholders is crucial to improve supportive care. In addition to cancer survivors themselves, healthcare professionals (HCPs) of various disciplines, play a critical role in defining and providing adequate supportive care after cancer treatment. These disciplines include medical specialists, general practitioners, occupational physicians and psychologists. In addition, informal caregivers, such as family caregivers and support consultants, have a large role in providing supportive care [23,25–27]. Insight in both the patients’ and HCPs’ perspectives is needed for adequate shared decision making in supportive care during and following treatment [28]. A previous study focusing on melanoma survivorship care [29] showed that HCPs considered the current supportive care offer limited and emphasized the necessity for personalised care. Although other studies have examined HCPs’ perspectives on improving supportive care for specific cancer types [29–31], in-depth insights into the perspective of a diverse group of HCPs, considering different professions and disciplines, and transcending cancer specialisations, are still lacking.

In light of the evolving landscape of cancer survivorship care, in which there is increasingly attention for cancer survivorship care, using a transdiagnostic approach to supportive care may be valuable. This approach allows focusing on the exploration of underlying needs and interventions transcending individual diagnoses, in this case cancer types [18,19]. The advantage of such an approach is the relevance to a larger group of cancer survivors, while also focusing on specific individual needs, in line with the trend towards more personalised care [18,19].

Therefore, the aim of this study was to explore the perceptions of cancer survivors and HCPs regarding adequate supportive care to cancer survivors following cancer treatment from a transdiagnostic perspective. Unlike previous qualitative studies that primarily describe supportive care needs in cancer survivorship, the present study also focuses on perceptions of how supportive care should be organized, implemented and integrated in clinical care practice, from both cancer survivors’ and multidisciplinary HCPs’ perspectives. The findings may serve as a foundational framework for the development of personalised supportive care tailored to the diverse and expanding population of cancer survivors.

## Materials and methods

### Study design

A qualitative study was conducted to gain an in-depth understanding of perceptions on adequate supportive cancer care [32]. We conducted individual interviews with cancer survivors and focus groups with oncological HCPs from different disciplines. This study is part of a larger project on resuming life after cancer [14]. To explore the perceptions of cancer survivors, we considered individual interviews most appropriate because of the sensitivity of the topic [33]. Perceptions of HCPs were explored using focus groups because of the expected positive effect of the interaction between participants and the expected lower sensitivity of the topic for this group of participants.

The Consolidated Criteria for Reporting Qualitative Research (COREQ) [34] were followed to correctly report the study, see S1 Appendix. The Ethics Review Board of the School of Social and Behavioral Sciences of Tilburg University approved this study [RP576] on 5 July 2021, as well as the Board of Directors of the Elisabeth-TweeSteden hospital [35], where this study took place.

We used a transdiagnostic approach in exploring perceptions towards adequate supportive care after cancer treatment. This approach, originating from mental healthcare, preserves underlying diagnosis and treatment while seeking to elucidate processes or develop interventions that are relevant to one or more diagnoses [18]. We explored the perceptions and needs of cancer survivors with various cancer types regarding supportive care, as well as the perceptions of HCPs from different professions and specializations, focusing on general and overarching perceptions and needs regarding supportive care.

### Selection of participants

For the interviews, cancer survivors were eligible for inclusion if they had been treated with curative intent and had a favorable prognosis after treatment. We used purposive sampling and aimed for variation among patients’ type of diagnosis (for example, breast cancer, colorectal cancer, and melanoma); sex; educational background; nationality; and time after diagnosis (ranging from 0 to 7 years). We aimed to mainly recruit cancer survivors in the age of 18–67 years, because we wanted to explore perspectives on resuming work as well. Eligible patients were recruited by their attending physician who provided them a patient information letter about the study at their treating hospital (the Elisabeth-TweeSteden Hospital (ETZ) in Tilburg in the South of the Netherlands). Participants for the interviews were recruited from 6 September 2021 to 9 March 2022, participants for the focus groups were recruited from 30 May 2022 to 22 September 2022.

For the focus groups, HCPs were eligible for inclusion if they had experience with providing care to patients with cancer. We used purposive sampling [33] to include a variable sample of oncological HCPs in terms of their discipline (including medical specialists, nursing specialists, general practitioners, occupational physicians, social workers, psychologists and support consultants) and specialization, as well as in terms of sex. In addition, for each focus group, we aimed for mixed samples of oncological HCPs. To recruit HCPs, invitations for participation were shared with associations for health psychologists, occupational physicians, general practitioners and medical specialists. Furthermore, the networks of the involved researchers and healthcare practitioners were used to approach HCPs, using the criteria listed above.

For both the interviews and the focus groups, potential participants could sign up through an online Microsoft Forms in which they were asked for contact information, background characteristics, and consent to participate. Interviewees were offered a gift card of €25 in exchange for their participation and focus group participants were offered a small gift (chocolate bar). There was no dropout. After interviews with 22 cancer survivors, and four focus groups with a total of 25 HCPs, thematic saturation (see Data analysis) [33,36] was reached and recruitment of participants ended.

### Data collection

Semi-structured interviews with cancer survivors were conducted face-to-face at a location of the participant’s preference (at Tilburg University or the participant’s home), or online (via Microsoft Teams). One of the researchers (DvdS), a senior researcher with a background in health communication sciences and experience in conducting interviews, conducted the interviews, which lasted approximately 60 minutes. This researcher was not previously acquainted with the interviewees. A predefined interview topic guide (S2 Appendix), based on previous literature, experience of the research group and input of patient experts was used to conduct the interviews [10,37,38]. It consisted of two parts: 1) Part 1 addressed cancer survivors’ experiences with resuming their life after treatment, of which the results are described in another qualitative interview study [14], 2) Part 2 focuses on perceptions towards comprehensive transdiagnostic supportive care including supportive care needs (current study). The interview guide was pilot tested with a patient expert and consequently improved.

The focus groups with HCPs were conducted online via Microsoft Teams. The focus groups, each lasting approximately 90 minutes, were moderated by MJ (endowed professor) and DvdS (senior researcher) with experience in facilitating focus group discussions. A topic guide based on previous literature was used to structure the focus groups [10,29,37] addressing perceptions on offering comprehensive transdiagnostic supportive care (S3 Appendix). Before starting the focus groups, the moderators explicitly explained that no consensus had to be reached. Moreover, the focus group moderators ensured that all participants were given the opportunity to speak during the focus groups. The topic guide for HCPs was not pilot tested, however, it was checked by co-authors who are HCPs themselves (DKW, LVB).

The interviewer and focus groups moderators were experienced qualitative researchers and were aware of possible biases because of their professional backgrounds in psychosocial oncology. Reflexivity was addressed through memo-writing, team discussions and independent coding checks. Participants could sign up for participation in the study if interested, there was no drop-out after signing up.

### Data analysis

The 22 interviews and 4 focus groups were audio recorded and transcribed verbatim without identifiers. The interview and focus group data from cancer survivors and HCPs were analysed separately. This approach allowed preservation of stakeholder-specific perspectives and meaning structures before comparing convergence and divergence between groups in the interpretation phase. Although the datasets were analyzed separately, a similar analytic process was used. A thorough inductive thematic content analysis drawing on elements from grounded theory was applied [39–41]. This included the cyclical process of data collection and analysis, using multiple phases of coding, constant comparison [33,42], and sampling until saturation [39–41,43,44].

More specifically, the analysis process consisted of the following steps. After each session, one of the researchers (DvdS) made a short summary. Second, the transcripts of four interviews and two focus groups were read, re-read and openly coded by one researcher (DvdS) and checked by a second researcher (ML, YAR, MJ) [39,41]. Next, an initial coding tree was developed from the unstructured open codes, leading to initial concepts and sub-concepts. Using this coding tree, the remaining transcripts were simultaneously openly and axially coded [39,41] by two researchers and complemented by additional codes if necessary [45]. The coding tree was constantly adapted during this coding phase. In the next phase (selective coding), linkages were identified between the (sub)concepts. Lastly, main- and subthemes were identified, and the coding tree was finalized, see S4 Appendix. All phases of the analysis process were extensively discussed in an interdisciplinary team of researchers (i.e., the authors). The software program ATLAS.ti 22 was used to analyze the transcripts.

Thematic saturation was assessed by examining both code saturation (no new codes emerging) and meaning saturation (no new dimensions within existing themes). After analyzing 22 interviews and 4 focus groups, no new codes or meanings were identified, and saturation was considered reached [33,36]. Two patient experts provided feedback on the findings and agreed with the themes that emerged. The findings of the focus groups were checked by co-authors who are HCPs themselves (DKW, LVB), and they agreed with the themes that emerged.

## Results

### Participant characteristics: cancer survivors

Of the 22 participants, 16 were female, and the mean age was 52.7 years (range 23–77 years) (see Table 1). Most participants (n = 13) were currently working, and others were partly on sick leave (n = 3), fully on sick leave (n = 3), unemployed (n = 2) or retired (n = 1). Participants were diagnosed with various cancer types and were treated with various treatments. Most participants completed their treatment (n = 16) and 6 participants were in long-term treatment such as immunotherapy or hormone therapy.

**Table 1:** Participants’ characteristics: cancer survivors.

ID	Sex, Age	Currently working	Diagnosis	Years after diagnosis*	Finished or current treatments	Time since finishing primary treatment
P1	F, 43	Partly on sick leave	Breast cancer with metastases	0–2	Finished: surgery, chemotherapy, radiation Current: hormone therapy	10 months
P2	F, 52	Fully at work	Breast cancer	3–4	Finished: surgery, radiation	4 years
P3	F, 59	Fully at work	Breast cancer	0–2	Finished: surgery, radiation	11 months
P4	F, 67	Fully at work	Breast cancer	0–2	Finished: surgery, radiation	2 years
P5	M, 53	Fully on sick leave	Laryngeal cancer with metastases	0–2	Finished: surgery, chemotherapy, radiation, chemoradiation	4 months
P6	F, 59	Fully at work	Breast cancer	3–4	Finished: surgery, radiation	3 years
P7	M, 64	Fully at work	Colorectal cancer	3–4	Finished: surgery	2 years
P8	F, 61	Unemployed	Colorectal cancer	0 to 2 and 3–4	Finished: 2 surgical interventions	4 years
P9	M, 46	Partly on sick leave	Colorectal cancer	0–2	Finished: surgery, chemotherapy	3 months
P10	F, 58	Fully at work	Colorectal cancer	3–4	Finished: surgery	3 years
P11	M, 53	Unemployed	Stomach cancer	0–2	Finished: surgery, chemotherapy	4 months
P12	F, 52	Fully at work	Stomach cancer	0–2	Finished: surgery	1 year and 10 months
P13	F, 42	Partly on sick leave	Colorectal cancer	0–2	Finished: surgery	8 months
P14	M, 44	Fully at work	Colorectal cancer	3–4	Finished: surgery, chemotherapy	1 year and 9 months
P15	F, 46	Fully at work	Breast cancer and thyroid cancer	0–2	Finished: surgery, chemotherapy, radiation Current: hormone therapy	8 months
P16	F, 77	Retired	Melanoma	0 to 2 and 3–4	Finished: surgery	7 years
P17	F, 48	Fully on sick leave	Thyroid cancer	0–2	Finished: surgery Current: iodine treatment	9 months
P18	F, 49	Fully at work	Melanoma	0–2	Finished: surgery	10 months
P19	F, 23	Fully on sick leave	Thyroid cancer	0–2	Finished: surgery Current: iodine treatment	7 months
P20	M, 53	Fully at work	Melanoma	7	Finished: 4 surgical interventions	7 years
P21	F, 69	Fully at work	Melanoma	0–2	Finished: surgery Current: immunotherapy	8 months (currently on treatment)
P22	F, 41	Fully at work	Melanoma	0–2	Finished: radiation Current: immunotherapy	3 months (currently on treatment)

*This range was used to classify the time after diagnosis.

### Participant characteristics: healthcare professionals

Of the 25 participants, 16 were female, and the mean age was 48.6 years (range 29–71). HCPs’ professions included: medical specialists, general practitioners, psychologists, occupational physicians, support consultants oncology, nursing specialists oncology and medical social workers. Table 2 presents an overview of characteristics of HCPs.

**Table 2:** Participants’ characteristics: healthcare professionals.

**Focus group number**	**Participants n**	**Age range**	**Female**	**Profession (n)**
1	7	36-59	5 (71%)	- Internist-oncologist (1) - General practitioner (1) - Health psychologist (2) - Occupational physician (oncology specialisation) (1) - Support consultant oncology (2)
2	6	29-57	5 (83%)	- Oncologist (1) - Gynaecologist (1) - Nursing specialist oncology (1) - Health psychologist (2) - Occupational physician (oncology specialisation) (1)
3	6	50-71	4 (66,7%)	- Oncologist (1) - Gastrointestinal surgeon: specialized in esophageal and gastric cancer (1) - General practitioner (2) - Occupational physician (oncology specialisation) (1) - Medical social worker (1)
4	6	33-62	2 (33,3%)	- Support consultant oncology/ oncology nurse (1) - Oncological surgeon: particularly of breast, skin, and colon (1) - Surgeon specialized in brain tumor surgery (1) - Oncologic surgeon: breast cancer, soft tissue tumors, melanoma, thyroid (1) - General practitioner (1) - Occupational physician (1)

### Perceptions on adequate supportive care after treatment of cancer

We identified 2 main themes with 7 underlying sub-themes for cancer survivors and 3 main themes and 8 underlying subthemes for HCPs, regarding their perceptions on adequate supportive care after treatment of cancer. The main themes and subthemes of both groups, which partly overlap, are presented in Table 3 and described in detail below.

**Table 3:** Overview of main themes and subthemes for cancer survivors and HCPs regarding their perceptions on adequate supportive care after treatment of cancer.

Cancer survivors:
1. Need for human-centered approach to supportive cancer care offered across the disease trajectory 1.1 Being recognized as a human being rather than a patient 1.2 Supportive care tailored to personal values and needs 1.3 Extended tailored support of healthcare professionals across the disease trajectory
2. Need for broader and easily findable and accessible psychosocial care 2.1 Broader (non-medical) psychosocial care including peer support 2.2 Specific support in maintaining or returning back to work 2.3 Extending psychosocial support to close relatives 2.4 Easily accessible and tailored follow-up care
**Healthcare professionals:**
3. Need for a personalized approach in cancer care with a focus on cancer survivors’ empowerment 3.1 Using a person-centered approach in supportive cancer care 3.2 Promoting cancer survivors’ empowerment after treatment and supporting them in finding balance and control over their changed lives
4. Extending content of supportive cancer care with reliable psychosocial care options 4.1 Need for broader psychosocial care 4.2 Need for specific attention for return to work
5. Towards integrated, collaborative supportive care 5.1 Awareness of the importance of timely supportive care among oncological HCPs 5.2 Knowledge on, and an overview of, reliable supportive care referral options 5.3 Adequate embedment of supportive care in clinical care and availability of sufficient resources 5.4 Clear role division and adequate multidisciplinary collaboration

### Perceptions of cancer survivors on appropriate supportive cancer care

1 **Need for human-centered approach to supportive cancer care offered across the disease trajectory**

The first main theme from the perspective of cancer survivors concerned the need for a human-centered approach to supportive cancer care offered across the disease trajectory, consisting of three subthemes: 1.1) Being recognized as a human being rather than a patient, 1.2) Supportive care tailored to personal values and needs, 1.3) Extended tailored support of HCPs across the disease trajectory.

1.1 *Being recognized as a human being rather than a patient*

Cancer survivors emphasized the importance of a human-centered approach from HCPs, where they are recognized as human beings rather than just patients. This entailed HCPs recognizing patients as unique individuals including their values, goals, personality and preferences. Cancer survivors preferred HCPs who demonstrate empathy by listening to their stories, inquiring about their feelings, and making a genuine effort to really understand their personal situation.

*Sub-theme 1.1:* Being recognized as a human being rather than a patient:

*“Certainly when you have cancer and certainly when your life is very uncertain and you are on a fast train [of treatments] (…) I think it is very important that you are seen as yourself and not as a number.” (P22; woman, age 41, melanoma)*

1.2 *Supportive care tailored to personal values and needs*

In addition to wanting to be seen as a human being, cancer survivors emphasized the need for supportive care to align with their personal values and needs. This implies that the provision of supportive care must be tailored not only to their disease characteristics, but also to what they consider important in life, which may also change over time. Consequently, needs may change overtime as well. According to survivors, it requires HCPs who are sensitive to this and be able to assess whether, when, and what kind of supportive care a patient needs. Some survivors also highlighted the importance of HCPs adjusting their communication to meet the specific needs of cancer survivors, for example by dosing the amount of information provided or by using a more positive, a humorous, or a more serious tone of voice.

*Sub-theme 1.2:* Supportive care tailored to personal values and needs:

*“It is important that they [HCPs] can read very well who they are dealing with. (…) That they can at least read what the patient is like, the character. That they are not too serious in the communication, in my case. With another patient, they may have to be very serious, because he prefers so. The HCP should sense this. ….” (P5; Male, age 53, larynx cancer)*

*Sub-theme 1.2:* Supportive care tailored to personal values and needs:

*“I think it needs to be properly asked to someone if it is [the supportive care] still appropriate? Because needs change over time. It should be properly checked every once in a while, whether expectations are still the same, whether needs are different or not, or if more or less is needed? (P13; Female, age 42, colorectal cancer)*

1.3 *Extended tailored support of HCPs across the disease trajectory*

Cancer survivors indicated that supportive care, such as psychosocial, work-related or spiritual support, should be offered across the entire disease trajectory, including shortly after the diagnosis, at multiple times during the disease trajectory, and after finishing treatment. They particularly stressed needing extended support after treatment. They generally experienced support from HCPs during treatment in terms of both medical care and supportive guidance. However, after treatment, when the number of appointments with HCPs decreased, survivors generally missed support of their HCPs. Some felt they were thrown in the deep end after treatment. Although some cancer survivors felt no current need for supportive care, the availability of supportive care in case they would need it later, provided them with a feeling of rest.

*Subtheme 1.3*: Extended tailored support of HCPs across the disease trajectory:

*“Supportive care should be offered at multiple times, (….). So at the beginning of the trajectory, but also halfway through the trajectory. In a lot of cases, cancer is treated with surgery as well as post-treatment, so somewhere between those two treatments you would want it again. (…) And maybe at some point two months after the treatment.” (P12; woman, age 42, colon c*ancer)

2 **Need for broader and easily findable and accessible psychosocial care**

The second main theme identified among cancer survivors was the need for broader and easily findable and accessible psychosocial care. This theme consisted of four sub-themes: 2.1) Broader (non-medical) psychosocial care including peer support, 2.2) Specific support in maintaining or returning back to work, 2.3) Extending psychosocial support to close relatives, and 2.4) Easily accessible and tailored follow-up care.

2.1 *Broader (non-medical) psychosocial care including peer support*

Besides medical supportive cancer care, cancer survivors indicated a need for psychosocial care and guidance in how to resume their life after treatment. Many survivors experienced a lack of communication about options for psychosocial supportive care from their HCPs. Instead, they had to seek psychosocial supportive care by themselves. As for the type of psychological support, cancer survivors highlighted the need for in-depth conversations about their feelings and experiences with the disease and with resuming life. This included a focus on dealing with the impact of the disease and its treatment on several life domains, considering their unique situation and personality. Some survivors also valued guidance in setting goals, for instance in terms of their lifestyle or in returning back to work. The need for reliable information about alternative medicine options was also mentioned, something medical HCPs rarely addressed.

*Sub-theme 2.1:* Broader (non-medical) psychosocial care including peer support:

*“Yes those are things [walking stairs, cycle etc] that I can do, but there are many more areas in my life, that I as a working mother had to take care of (...) How do I find the balance? How can I deal with cancer, considering my character and personality? So then they referred me to a psychologist, who is also specialized in this.” (P1, woman, age 43, breast cancer)*

The need for peer support was particularly emphasized by cancer survivors. They indicated that exchanging experiences and advice with peers who also have or have had cancer leads to recognition and understanding. They felt strengthened and supported by realizing they were not the only ones. Some preferred peers with the same type of cancer and/or age. Having gained so much from peer support themselves, survivors sometimes wanted to provide peer support to others to share their experiences and to be able to help others as well. However, some cancer survivors did not prefer peer support, as they wished to avoid being confronted with the disease and potentially distressing stories from others. Others indicated that talking to a loved one with cancer in their own social network provided them with the support they needed.

*Sub-theme 2.1:* Broader (non-medical) psychosocial care including peer support:

*“We were all sitting together in the waiting room; one lady has lost her hair, another is in a wheelchair, another is very thin because of the treatments. We were all sitting there with something. I think that this gives you the strength to say, okay, if they can do it, then I can do it too.” (P22; woman, age 41, melanoma)*

2.2 *Specific support in maintaining or returning back to work*

Cancer survivors indicated to experience a need for (practical) guidance in maintaining or returning back to work. They lacked insight in regulations (from the government) during sick-leave, and preferred support and information about rules and regulations during and after sick-leave. For instance, in the Netherlands, after two years of sick leave, the employer is allowed to fire the employee. According to survivors, a lack of clarity about the rules, regulations, and subsequent steps in the process led to feelings of insecurity. Furthermore, they indicated the need for information regarding financial support options, social benefits and support. Furthermore, they preferred support in returning to work or maintaining work. They indicated a need for support in communication with their employer, tips for how to resume their work, or support in finding new work that better fits their sometimes changed abilities.

*Subtheme 2.2:* Specific support in maintaining or returning back to work:

*“So in a few months my ‘two years of illness’ are over and my employer may fire me after this. The next step is a sickness pay arrangement. (…) So I'm trying to figure out how I could arrange this with my employer, so that my quality of life remains the same. I'm not someone who has to sit at home all the time. I think I can really contribute. But now I still feel that I don’t get the support and that the rules are just the rules.” (P1; Woman, age 43, breast cancer)*

2.3 *Extending psychosocial support to close relatives*

Cancer survivors emphasized the importance of HCPs being aware of the potential impact of the disease on partners and close relatives, who often experience significant emotional strain and may struggle to cope. Particularly as close relatives often provided practical and emotional support to the patient. Therefore, they stressed the importance of extending the provision of psychosocial support to their close family members, to help them learn to cope with the effects of their loved one’s illness.

*Subtheme 2.3:* Extending psychosocial support to close relatives:

*“My wife had a severe burnout after I was sick. She had to take care of me for months and I couldn't do anything. She spent those months under severe stress, taking care of the children (...). Then she really had a psychological dip, she developed a trauma (...) and then she sat at home for six months. She visited a psychologist for that, and got medication to keep herself calm. In that respect her aftermath has been heavier than my aftermath. You have to remember that the partner has to endure it just as hard, maybe even harder, and has to face everything. That was not easy for her.” (P14; Male, age 44, colorectal cancer)*

2.4 *Easily accessible and tailored follow-up care*

Cancer survivors felt a need for their HCPs to be easily accessible and available for follow-up care. For instance, they valued a phone number to contact a healthcare professional for support with a low threshold. Even if they did not feel a need for supportive care at the moment of the interview, the idea of having the opportunity to get support whenever needed provided them with a sense of security. Most survivors valued timely follow-up appointments to check for possible recurrence of the disease because of their fear of recurrence. They also indicated that follow-up care should be tailored to their specific needs, for example by having the opportunity for an additional and sometimes earlier than scheduled follow-up appointment when needed.

*Subtheme 2.4:* Easily accessible and tailored follow-up care:

*“I can always call if I think something is wrong and then I can come in pretty quickly. Because I've had it [the disease] several times I stay under control all my life. (...) At one point the doctors said, you can also go to an annual check-up, but I don't want that, then I won't have peace of mind, so I can come every six months. I think a year is a very long time, I don't want that and they agree. It's nice that I can tell what I want, that gives me peace of mind.” (P20; Male, age 53, melanoma)*

### Perceptions of HCPs on appropriate supportive cancer care

3 **Need for a personalized approach in cancer care with a focus on cancer survivors’ empowerment**

The third main theme identified, and the first main theme among HCPs, was the need for a personalized approach in cancer care with a focus on cancer survivors’ empowerment. This theme consisted of two sub-themes: 3.1) Using a person-centered approach in supportive cancer care, and 3.2) Promoting cancer survivors’ empowerment after treatment and supporting them in finding balance and control over their changed lives.

3.1 *Using a person-centered approach in supportive cancer care*

HCPs stressed the need to use a personalized approach during and after treatment when anticipating on supportive cancer care. They explained that personalized supportive care – meaning a tailored approach based on survivors’ personal characteristics, needs and values – is essential. The personal values and goals of patients can change over time, and consequently their needs regarding medical as well as supportive care vary. HCPs also highlighted that due to an increased survival rate, an increased number of young cancer survivors face specific challenges such as work- and parenthood related issues. Therefore, adjusting supportive care to personal values and needs of survivors is very essential according to HCPs. To facilitate personalized supportive care, HCPs considered it important to encourage cancer survivors to reflect on what matters to them, as each individual has unique needs and may not require (every type of) supportive care. Moreover, HCPs think that the individual survivors’ values and preferences with survivors themselves should be discussed as an important starting point of providing personalized supportive care, rather than just discussing treatment options in a multidisciplinary team of HCPs.

*Sub-theme 3.1:* Using a person-centered approach in supportive cancer care:

*“I think that the role of a medical specialist lays in recognizing certain things, so that you ask questions about supportive care needs and then recognize the needs. I don't think that offering something structural would be good, because I also see plenty of patients who resume their life more easily and who have a lot of support from loved ones, from family and friends and manage with that as well. But I think mainly awareness and recognizing that, is important.” (P6; Male, age 39, internist-oncologist)*

3.2 *Promoting cancer survivors’ empowerment after treatment and supporting them in finding balance and control over their changed lives*

HCPs reported that cancer survivors often struggle to regain control over their lives as they seek to find a new balance and directions after treatment. After a long treatment trajectory with intensive support from HCPs, where HCPs typically lead the process, survivors must resume their life and find appropriate support on their own. Many cancer survivors feel inadequately empowered and unprepared for this transition, according to HCPs. HCPs noted that survivors may face fear of recurrence of cancer and fear of death, and are often overwhelmed with emotions, sometimes leading to an emotional fall out episode. Therefore, HCPs emphasized the importance of preparing survivors for the potential impact of the disease and treatment on their lives. They stressed the need to support survivors in finding a new balance and regaining control during and after treatment. To achieve this, it is crucial to validate survivors’ feelings and to provide tools on how to cope with all the changes they face.

*Subtheme 3.2:* Promoting cancer survivors’ empowerment after treatment and supporting them in finding balance and control over their changed lives:

*“After treatment, the patient literally goes from surviving to living again, but that life is going to look different. When patients say they want to become their old selves again, I always say - although I find it very confronting – that they are not going to become that again, because too much has changed. So I do warn for that. If patients think, they will soon be able to do everything exactly as before, that's just not realistic.” (P22; Female, age 54, occupational physician)*

HCPs emphasized the importance of promoting cancer survivors’ empowerment and supporting them in regaining balance and control over their changed lives. According to HCPs, survivors’ self-reliance tends to increase when their treatment ends, necessitating the promotion of empowerment both during and after treatment. Patient empowerment may enhance survivors’ understanding of their own needs and the ability to communicate these to HCPs, aiding in the identification of appropriate supportive care. This was perceived as beneficial given the current limited capacity of HCPs.

*Sub-theme 3.2:* Promoting survivors’ empowerment after treatment and supporting them in finding balance and control over their changed lives:

*“We give people a referral guide, which is compiled by us, with different life domains in it, containing information about nutrition, energy management, life questions, addresses of general practitioners and reliable websites. So, we provide a selection of sources where they can find information. (…) We want to empower people to get started themselves, so that when the time comes, that they can look at it for the first information.” (P1; woman, age 53, Support consultant oncology)*

*Sub-theme 3.2:* Promoting survivors’ empowerment after treatment and supporting them in finding balance and control over their changed lives:

*“What I think is a good discussion, is whether it's right that the direction lies or should lie with the medical specialist or healthcare professional, because it's not possible to keep track of everything that is going on with the patient. So I think, on the contrary, the direction should lie more with the patient, so that the patient can also inform the healthcare professionals better and ask more specific and better care questions.” (P10; woman, age 29, Oncology nursing specialist)*

4 Extending content of supportive cancer care with reliable psychosocial care options

The fourth main theme identified, and the second one among HCPs, was extending the content of supportive cancer care with reliable psychosocial care options. Within this theme, two subthemes were identified, 4.1) The need for broader psychosocial care, and 4.2) The need for specific attention for return to work.

4.1 *Need for broader psychosocial care*

To be able to meet cancer survivors’ varying needs, HCPs reported the importance of broader psychosocial care that extends beyond traditional medical care and in which attention is paid to all life domains considered relevant to individual survivors. This included attention for the impact of cancer on family life and work, but also on social and romantic relationships. Furthermore, according to HCPs, specific support for adolescents and young adults with cancer is necessary, as they experience specific problems related to (returning to) work, and dealing with the impact on children and/or desire to have children. They also mentioned the value of peer support, preferably in moderated (online) patient platforms, as peer support may provide recognition for cancer survivors. Furthermore, the value of trustworthy alternative care was mentioned as well as centers for information and support in cancer that should be brought to survivors’ attention.

*Sub-theme 4.1:* Need for broader psychosocial care:

*“It may surprise you, what I miss most is a list of good alternative practitioners. Because I know, a lot of patients have a need for it, for instance acupuncture for hormonal problems (…) because what I have to offer is a lot of intensive medical treatment or is high risk. (…) So I would like to have a list with good reliable alternative practitioners who are affiliated with professional organizations, (…) that's what I might miss the most.” (P9; woman, age 55, gynecologist)*

4.2 *Need for specific attention for return to work*

HCPs highlighted the importance of specific attention to survivors’ return to work. According to HCPs, survivors’ abilities to return to work vary significantly and the challenges they may face are often unpredictable. Several issues impeding the return-to-work process were identified, especially among younger cancer survivors. These include dealing with career interruptions, returning to work while exceeding personal capacities, and dealing with the lingering effects of their disease. One key reason for exceeding their capabilities, according to HCPs, was survivors’ attempt to fulfil the expectations of their social network after treatment. HCPs added that whenever survivors were unable to fulfill these expectations, they often felt unheard and misunderstood.

Furthermore, several HCPs indicated that employers and occupational physicians sometimes put pressure on cancer survivors who may not be ready to return to work yet. Conversely, they also indicated that employers and occupational physicians might hinder survivors eager to return to work because of perceived risks or challenges associated with employing them [46]. HCPs also stressed that survivors often have limited control over their own sick leave process because of inflexible laws and regulations that do not accommodate the specific needs of survivors in return to work.

HCPs thought that survivors should be encouraged in a positive way to resume their work. HCPs believed that returning to work should be part of the medical treatment plan, with the involvement of an occupational physician early in the treatment process. This plan should include preparations for the potential impact of the disease and the treatment on both life and work. HCPs perceived constructive support of (oncological specialized) occupational physicians as crucial, given their greater capacity and expertise for intensive counseling of survivors compared to medical specialists.

*Sub-theme 4.2:* Need for specific attention for return to work:

*“There is still a lot of unawareness with employers in how to deal with their employee who has cancer and consequently patients are left alone for a long time. (…) When the treatment is finished, the employee is in the deepest valley of his working capacity, he is in the worst shape in terms of work, and then reintegration starts. (…) Then pressure arises from the work system and from laws and regulations.” (P7; woman, age unknown, occupational phy* *sician (oncology specialization)*

5 **Towards integrated, collaborative supportive care**

The fifth main theme identified, and the third one among HCPs, was enhancing integrated, collaborative supportive care, consisting of four subthemes, 5.1) Awareness of the importance of timely supportive care among oncological HCPs, 5.2) Knowledge on, and an overview of, reliable supportive care referral options, 5.3) Adequate embedment of supportive care in clinical care and availability of sufficient resources, and 5.4) Clear role division and adequate multidisciplinary collaboration.

5.1 *Awareness of the importance of timely supportive care among oncological HCPs*

HCPs emphasized the need for increased awareness of the importance of supportive care among HCPs in general, including awareness of the added value of oncological specialized occupational physicians. HCPs indicate insufficient awareness of the importance of supportive care due to guidelines that primarily focus on medical aspects and therefore overlook supportive care. In addition, HCPs stated that medical education rarely covers supportive cancer care adequately.

HCPs believe that supportive care is frequently underutilized and insufficiently referred to by medical HCPs in general, also stressing the need for raising awareness on its importance. They also stressed the importance of raising awareness on offering timely supportive care. They stated that currently, providers of supportive care see cancer survivors at various stages of their medical trajectory, but often when problems have already occurred. HCPs suggested that many of these problems could potentially have been prevented by providing timely supportive care. To achieve this, HCPs recommended discussing supportive care including return to work during treatment or even at the first consultation. Additionally, HCPs reported that offering timely supportive care may help survivors to deal with a potential emotional fallout episode after treatment.

*Sub-theme 5.1:* Awareness of the importance of timely supportive care among oncological HCPs:

*“I see that people stay away from the workplace for far too long. Of course not everyone can work, but if the gap becomes too big, the reintegration is often unbridgeable and people have to deal with the cancer and everything else that comes with it, but they also have to make an effort in their work to show that they really can do it again, and then they cross their boundaries. As an oncology occupational physician consultant, patients are referred to me from all over the country, a year and a half or 2 years after diagnosis, and they say: Why couldn't I have had this support much earlier?” (P8; Woman, age 57, Occupational Physician Oncology)*

*Sub-theme 5.1:* Awareness of the importance of timely supportive care among oncological HCPs:

*“I notice now that there is a whole world around it and that there is actually far too little attention to it [supportive care], even within primary care, so yes, that is my motivation to participate in this focus group.” (P14; woman, age 53, general practitioner)*

5.2 *Knowledge on, and an overview of, reliable supportive care referral options*

HCPs highlighted the need for improved knowledge and a structured overview of reliable supportive care referral options, particularly for options outside the hospital, such as informal and oncological specialized supportive care. They stressed the importance of HCPs being knowledgeable about occupational physicians who specialize in return to work after cancer, as the current lack of awareness impedes their referral of cancer survivors appropriately.

HCPs expressed a preference for a structured overview of available reliable and trustworthy supportive care referral options, including options of psychosocial care both regionally and nationally. They suggested that patient associations and oncology networks could significantly aid in providing or directing supportive care. Furthermore, HCPs indicated a need for a tool or application to assist HCPs in the assessment and referral process to ensure that survivors receive timely and adequate supportive care. This tool would help to streamline the referral process and ensure that HCPs can effectively connect survivors with the appropriately supportive care options.

*Sub-theme 5.2:* Knowledge on, and an overview of, reliable supportive care referral options:

*“I get a lot of folders sent to me and also via social media, and in that respect it would be helpful if there was a ‘preferred provider’, so that in the region I could refer to these reliable providers, and then I can experience how this works best in practice.” (P23; Male, age 62, general practitioner)*

5.3 *Adequate embedment of supportive care in clinical care and availability of sufficient resources*

HCPs underscored the necessity of integrating supportive care in the clinical care pathway and ensuring the availability of sufficient resources. They reported that a structured approach or standardized pathway for supportive care after cancer treatment is often lacking, which often leads to its ad-hoc provision. Therefore, HCPs believed that supportive care, including aspects such as return to work, should be systematically embedded in the clinical cancer care pathway for all cancer survivors.

Moreover, HCPs pointed out the constraints they face, such as limited resources and insufficient coverage by cancer survivors’ health insurances for multidisciplinary supportive care. HCPs also highlighted the financial challenges in hiring additional dedicated professionals to provide supportive care. Consequently, HCPs emphasized the need for improved resource allocation, including time and capacity, to effectively embed supportive care into clinical practice.

*Sub-theme 5.3:* Adequate embedment of supportive care in clinical care and availability of sufficient resources:

*“Clinical care pathways are made for anything and everything, but for the rehabilitation phase or the phase after treatment of cancer, no clinical care pathway is available.” (P17; Male, age 50, gastrointestinal surgeon)*

*Sub-theme 5.3:* Adequate embedment of supportive care in clinical care and availability of sufficient resources:

*“It’s important for GPs that we know what supportive care is available. (…) Most referrals are done via a medical system; it would be very convenient if the supportive care provider also makes use of that. Patients very often ask whether it will be reimbursed, and this depends per insurance, and if not, this is quite a barrier.” (P2; Male, age 59, general practitioner)*

5.4 *Clear role division and adequate multidisciplinary collaboration*

HCPs reported the need for clear role division and effective multidisciplinary collaboration in supportive care. They argued that it is often unclear to both cancer survivors and HCPs who can be consulted for specific problems after treatment, and they stressed that there is often ambiguity about the roles and responsibilities of various HCPs involved in supportive care. HCPs indicated that roles could be clarified by designating leading roles to specific types of HCPs in supportive care, such as treating physicians, nursing practitioners, specialized nurses, case managers or general practitioners. HCPs also emphasized the need for a designated point of contact to coordinate supportive care efforts, which would streamline referrals and improve communication among HCPs.

HCPs also suggested enhancing multidisciplinary collaboration, involving primary care, secondary care and informal care. According to the HCPs, effective collaboration requires better information exchange and alignment regarding referrals to ensure that all HCPs are aware of the cancer survivor's treatment progress and supportive care needs. The involvement of relatives, patient associations, and (regional) oncology networks in supportive care was emphasized as essential to improve support and multidisciplinary collaboration.

*Sub-theme 5.4:* Clear role division and adequate multidisciplinary collaboration:

*“Well, it would help if the roles were more clearly defined, that there was agreement and that it would be clear what can be expected of us [general practitioners]. (…) it would help enormously if it's clear what each HCP’s role is.” (P23; Male, age 62, general practitioner).*

## Discussion

### Summary of main findings

In this study we explored the perceptions of cancer survivors and oncological HCPs regarding adequate supportive cancer care after treatment.

Both groups shared the view that supportive care should be person-centered, tailored to individual values and needs, with an expansion of psychosocial care options, and particular attention to work-related issues. Survivors primarily emphasized what supportive care should entail, including being recognized as a human with own values, goals and preferences, rather than being perceived as ‘a patient’, including receiving holistic support across the disease trajectory. Furthermore, they emphasized valuing easily accessible care that aligns with their changing needs. In contrast, HCPs focused more on the conditions required to deliver person-centered supportive care in routine healthcare practice, including promoting patient empowerment, strengthening multidisciplinary collaboration, clarifying roles and responsibilities, and improving the integration of supportive care into clinical practice.

Combining the perceptions of both cancer survivors and HCPs, the findings suggest that improving supportive care requires both care that is responsive to survivors’ individual values, needs and preferences, as well as organizational changes that enable HCPs to provide and coordinate such care effectively.

### Interpretation of findings

The perceptions of both cancer survivors and HCPs highlight the need to expand psychosocial care, and focus supportive care on individual’s values, preferences, and needs using a person-centered approach, which contributes to improving their quality of life and long-term well-being. Specifically, a shift from patient-centered to person-centered care may be needed. While both approaches share similarities, their main goals differ: patient-centered care primarily aims to help individuals to lead functional lives by focusing on reducing suffering, whereas person-centered care is aimed at supporting meaningful lives by prioritizing what matters most to each individual [47,48]. In person-centered care, treatment and support are aligned with a person’s unique goals, values, and preferences across various life domains, such as work, relationships and personal identity; a deeper understanding of the whole person, beyond just the medical condition [47,48]. The values, goals and preferences guide all aspects of their care, in a dynamic relationship with relatives and HCPs [49]. Furthermore, patients are respected and empowered to be involved in medical decisions [47,48]. Person-centered care tends to be associated with positive outcomes for patients as well as professionals, including better meeting patients’ individual needs, and improved job satisfaction among HCPs [50]. The current study’s use of a transdiagnostic approach—exploring perspectives beyond specific diagnoses—allowed for this more holistic understanding.

Another important finding, is that HCPs stressed the importance of focusing on patient empowerment as a key element of personalizing care. They indicated that patient empowerment would help patients to gain more insight into their own values, needs and preferences, while supporting the transition from patient to self-reliant citizen. Stimulating patient empowerment can contribute to a more feasible healthcare system – something that is urgently needed given the growing healthcare demands, due to an aging population, an increasing number of people with chronic diseases [51] and healthcare staff shortages [52]. Furthermore, stimulating patient empowerment contributes to putting unique individuals in control of their treatment, care and life [52]. On the other hand, cancer survivors indicated to need accessible and tailored supportive care. Improving and facilitating the accessibility and tailoring of supportive care, may help to enhance patient empowerment of cancer survivors.

HCPs also emphasized the importance of a coordinated approach involving collaboration of HCPs from different medical and non-medical disciplines, and integration of supportive care into clinical care. To realize integrated supportive care in the clinical healthcare setting, increasing awareness of supportive care was perceived as important, as well as the need for knowledge on and overview of reliable supportive care options. The identified need for integration of supportive care within the clinical setting is in line with previous studies, which have shown that current supportive care following cancer treatment is often not offered in a personalized way within the clinical healthcare setting, and therefore fails to meet cancer survivors’ needs [23,25–27]. Similarly to HCPs, this need was also indicated by cancer survivors, as they emphasised needing broader and more personalized (non-medical) psychosocial care. The insights from the current study support the need for person-centered supportive care and adds to this that adequate collaboration, clear role division among HCPs, and the structural embedment of supportive care into clinical care, may improve the appropriateness of supportive care for cancer survivors across the disease trajectory.

### Strengths and limitations

The main strength of this study is the adoption of a transdiagnostic perspective, which provides insight into supportive care needs that transcend individual cancer diagnoses. Moreover, by combining the perspectives of both cancer survivors and HCPs, this study highlights their unique as well as overlapping views and preferences, while both groups put emphasis on specific aspects of supportive care. Although some of the needs may appear self-evident, they appear to be yet unmet and unaddressed in current healthcare practice. It is important to address these unmet needs in supportive care using a personalized – or even better – person-centered approach. To further examine the perspectives and needs of cancer survivors and HCPs transcending different cancer diagnoses, a systematic review of qualitative studies could be valuable.

In this study, we only included cancer survivors with a favorable prognosis, in order to gain insight into resuming life after treatment, in line with our research question. However, people with a palliative diagnosis also often face the challenge of resuming their life while living with cancer and may experience partly similar supportive care needs. They may have other or more specific (information) needs in addition to survivors with a favorable prognosis, such as end-of-life care and advance care planning [53], which may limit the generalizability of the results to persons with a palliative diagnosis. Future research could therefore explore the perceptions of supportive care among patients with a palliative diagnosis as well. Furthermore, in this study, participating cancer survivors were recruited by their medical specialist following pre-defined selection criteria. However, bias could have occurred, for instance by inviting patients with severe problems or only few problems, leading to extreme cases. To minimize this potential bias, we alternated data collection and analysis and allowed initial findings guide further sampling, resulting in a diverse sample.

### Implications for practice

To improve integrated collaborative person-centered supportive care, first, awareness and knowledge of supportive care options should be enhanced. This may be accomplished by developing (online) decision support tools to provide adequate supportive care. In co-creation with researchers, HCPs, patients and communication- and IT professionals, the authors developed an online tool, based on the results of the current study and a previous study on experiences with resuming life after cancer [14]. The online tool consists of three core elements: 1) a self-evaluation scan to explore one’s needs for supportive cancer care, 2) an overview of personalized supportive care options, and 3) providing support in shared decision-making with HCPs. The tool can be used by cancer patients, as well as by HCPs, to find reliable options for supportive care and to make referrals. The tool is called ‘Back on Track bij Kanker’ [54] (translation: Back on Track with Cancer) and can be visited via this link: www.backontrackbijkanker.nl. The development of the ‘Back on Track with Cancer’ tool demonstrates that the findings of this study were translated into practice, thereby supporting the implementation of person-centred supportive care.

Furthermore, enhancing clarity in role division in supportive care among HCPs was considered important to improve multidisciplinary collaboration. The starting point of supportive care should be, according to cancer survivors as well as HCPs, in the clinical setting by paying attention to supportive care already during the treatment phase. Also, embedding supportive care at the policy and organizational level was considered important to enhance the integration of supportive care into clinical pathways. Policy adjustments should focus on the provision of adequate resources for supportive care. The findings of this study can be used as input for the development of person-centered supportive care tailored to the diverse and expanding population of cancer survivors.

## Conclusion

While both cancer survivors and HCPs emphasized the importance of comprehensive, person-centered supportive care for cancer survivors, its implementation in clinical practice remains challenging. Key requirements, such as greater awareness of the importance of supportive care among HCPs, knowledge of reliable referral options and interdisciplinary collaboration, are often unmet. Efforts should focus on improving HCPs’ awareness and knowledge of supportive care options and integrating supportive care into clinical practice. The findings of this study informed the development of the online tool ‘Back on Track with Cancer’ [54]. This tool supports the identification of supportive care needs, provides personalized supportive care options, and facilitates shared decision making with HCPs. The findings of this study can contribute to integrated collaborative person-centered supportive care tailored to the diverse and expanding population of cancer survivors.

## Supporting information

S1 Appendix COREQ checklist. (PDF)

S2 Appendix Topic guide: interviews with cancer survivors. (PDF)

S3 Appendix Topic guide: focus groups with healthcare professionals. (PDF)

S4 Appendix Coding tree. (PDF)
