# Supplementary material for: Perceptions of cancer survivors and healthcare professionals on adequate supportive care after treatment of cancer: An in-depth qualitative study
Source: PLoS One. 2026 Aug 25;21(8):e0355113. doi: 10.1371/journal.pone.0355113 (PMC13505917; doi:10.1371/journal.pone.0355113)
Supplement: Supplementary file 1 [file pone.0355113.s001.pdf]

S1 Appendix. COREQ checklist.

COREQ checklist: "Perceptions of cancer survivors and healthcare professionals on adequate supportive care after treatment of cancer: an in-depth qualitative study"

**Domain 1: Research team and reflexivity**	**Question**	**Answer**
Personal Characteristics		
1. Interviewer/ facilitator	Which author/s conducted the interview or focus group?	Interviews: DvdS<br>Focus groups: DvdS and MJ
2. Credentials	What were the researcher's credentials? E.g. PhD, MD	PhD (DvdS)<br>PhD (MJ)
3. Occupation	What was their occupation at the time of the study?	Senior researcher (DvdS) and endowed professor (MJ).
4. Gender	Was the researcher male or female?	Females
5. Experience and training	What experience or training did the researcher have?	Both are experienced in conducting quantitative and qualitative research, and they have studied the impact of cancer on cancer survivors for multiple years.
Relationship with participants		
6. Relationship established	Was a relationship established prior to study commencement?	Interviews: There were no connections established prior to the study commencement.<br>Focus groups: To recruit healthcare professionals (HCPs), invitations for participation were shared with associations for health psychologists, occupational physicians, general practitioners and medical specialists: there was no prior relationship with these HCPs. Furthermore, the networks of the involved researchers and healthcare practitioners were used to approach HCPs, using the inclusion criteria.<br>For both the interviews and the focus groups, participants had no previous knowledge about the study.
7. Participant knowledge of the interviewer	What did the participants know about the researcher? e.g. personal goals, reasons for doing the research	The participants had no prior knowledge about the goals and reasons for doing the research of the researchers.
8. Interviewer characteristics	What characteristics were reported about the interviewer/facilitator? e.g. Bias, assumptions, reasons and interests in the research topic	The following characteristics were reported: The interviews were conducted by DvdS, senior researcher with a background in health communication sciences and she is experienced in conducting interviews. The focus groups were moderated by MJ (endowed professor) and DvdS, both have experience in facilitating focus group discussions.<br>During the interviews the researchers aimed to not express personal views and to listen to the input of the participants carefully.
**Domain 2: Study design**		
Theoretical framework		

S1 Appendix. COREQ checklist.

COREQ checklist: "Perceptions of cancer survivors and healthcare professionals on adequate supportive care after treatment of cancer: an in-depth qualitative study"

9. Methodological orientation and Theory	What methodological orientation was stated to underpin the study? e.g. grounded theory, discourse analysis, ethnography, phenomenology, content analysis	A thorough inductive thematic content analysis drawing on elements from grounded theory was applied [see ref 39-41].
Participant selection		
10. Sampling	How were participants selected? e.g. purposive, convenience, consecutive, snowball	For the interviews, cancer survivors were eligible for inclusion if they had been treated with curative intent and had a favorable prognosis after treatment. We used purposive sampling and aimed for variation among patients' type of diagnosis (for example, breast cancer, colorectal cancer, and melanoma); sex; educational background; nationality; and time after diagnosis (ranging from 0 to 7 years). We aimed to mainly recruit cancer survivors in the age of 18 to 67 years, because we wanted to explore perspectives on resuming work as well. Eligible patients were recruited by their attending physician who provided them a patient information letter about the study at their treating hospital (the Elisabeth-TweeSteden Hospital (ETZ) in Tilburg in the South of the Netherlands).<br><br>For the focus groups, HCPs were eligible for inclusion if they had experience with providing care to patients with cancer. We used purposive sampling to include a variable sample of oncological HCPs in terms of their discipline (including medical specialists, nursing specialists, general practitioners, occupational physicians, social workers, psychologists and support consultants) and specialization, as well as in terms of sex. In addition, for each focus group, we aimed for mixed samples of oncological HCPs. To recruit HCPs, invitations for participation were shared with associations for health psychologists, occupational physicians, general practitioners and medical specialists. Furthermore, the networks of the involved researchers and healthcare practitioners were used to approach HCPs, using the criteria listed above.
11. Method of approach	How were participants approached? e.g. face-to-face, telephone, mail, email	Interviews: Eligible patients were recruited by their attending physician who provided them a patient information letter about the study at their treating hospital (the Elisabeth-TweeSteden Hospital (ETZ) in Tilburg in the South of the Netherlands).<br>Focus groups: To recruit HCPs, invitations for participation were shared with associations for health psychologists, occupational physicians, general practitioners and medical specialists: there was no prior relationship with these HCPs. Furthermore, the networks of the involved researchers and HCPs were used to approach HCPs.

S1 Appendix. COREQ checklist.

COREQ checklist: "Perceptions of cancer survivors and healthcare professionals on adequate supportive care after treatment of cancer: an in-depth qualitative study"

12. Sample size	How many participants were in the study?	We conducted 22 interviews with cancer survivors, and four focus groups with 25 HCPs, including medical specialists, general practitioners, occupational physicians and informal caregivers.
13. Non-participation	How many people refused to participate or dropped out? Reasons?	Participants could sign up for participation in the study if interested, there was no drop-out after signing up.
Setting		
14. Setting of data collection	Where was the data collected? e.g. home, clinic, workplace	The interviews with cancer survivors were conducted face-to-face at a location of the participant's preference (at Tilburg University or the participant's home), or online (via Microsoft Teams).<br>The focus groups with HCPs were conducted online via Microsoft Teams.
15. Presence of non-participants	Was anyone else present besides the participants and researchers?	No
16. Description of sample	What are the important characteristics of the sample? e.g. demographic data, date	The interviewed cancer survivors varied in (time since) diagnosis, sex and age. Cancer survivors were eligible for inclusion if they had been treated with curative intent and had a favorable prognosis after treatment. We aimed to mainly recruit cancer survivors in the age of 18 to 67 years, because we wanted to explore perspectives on resuming work as well.<br>For the focus groups, HCPs were eligible for inclusion if they had experience with providing care to patients with cancer. Oncological HCPs varied in terms of their discipline (including medical specialists, nursing specialists, general practitioners, occupational physicians, social workers, psychologists and support consultants) and specialization, as well as in terms of sex. For each focus group, we aimed for mixed samples of oncological HCPs.
Data collection		
17. Interview guide	Were questions, prompts, guides provided by the authors? Was it pilot tested?	The interviews and focus groups were conducted using a semi-structured approach with pre-defined topic guides. The interview guide was pilot tested with a patient expert and consequently improved. The topic guide for HCPs was not pilot tested, however, it was checked by co-authors who are HCPs themselves (DKW, LVB).
18. Repeat interviews	Were repeat interviews carried out? If yes, how many?	No repeat interviews were carried out.
19. Audio/visual recording	Did the research use audio or visual recording to collect the data?	Yes, the interviews and focus groups were audio recorded.
20. Field notes	Were field notes made during and/ or after the interview or focus group?	Yes. After the sessions, one of the researchers (DvdS) made a short summary of the interviews and focus group.

S1 Appendix. COREQ checklist.

COREQ checklist: "Perceptions of cancer survivors and healthcare professionals on adequate supportive care after treatment of cancer: an in-depth qualitative study"

21. Duration	What was the duration of the interviews or focus group?	The interviews lasted approximately 60 minutes. The focus groups lasted approximately 90 minutes.
22. Data saturation	Was data saturation discussed?	Yes
23. Transcripts returned	Were transcripts returned to participants for comment and/or correction?	No. However, the researcher summarized answers of participants during the interview to ensure the answers were correctly understood.<br>For the interviews, two patients provided feedback on the findings and agreed with the themes that emerged. The findings of the focus groups were checked by co-authors who are HCPs themselves (DKW, LVB), and they agreed with the themes that emerged.
**Domain 3: Analysis and findings**		
Data analysis		
24. Number of data coders	How many data coders coded the data?	The transcripts of four interviews and two focus groups were read, re-read and openly coded by one researcher (DvdS) and checked by a second researcher (ML, YAR, MJ). Next, an initial coding tree was developed from the unstructured open codes, leading to initial concepts and sub-concepts. Using this coding tree, the remaining transcripts were simultaneously openly and axially coded by two researchers and complemented by additional codes if necessary. The coding tree was constantly adapted during this coding phase. In the next phase (selective coding), linkages were identified between the (sub)concepts. Lastly, main- and subthemes were identified, and the coding tree was finalized. All phases of the analysis process were extensively discussed in an interdisciplinary team of researchers (i.e. the authors).
25. Description of the coding tree	Did authors provide a description of the coding tree?	Yes, a coding tree is attached in the appendix (S4 Appendix).
26. Derivation of themes	Were themes identified in advance or derived from the data?	The themes were derived from the data.
27. Software	What software, if applicable, was used to manage the data?	The software program ATLAS.ti 22 was used to analyze the transcripts.
28. Participant checking	Did participants provide feedback on the findings?	For the interviews, two patients provided feedback on the findings and agreed with the themes that emerged. The findings of the focus groups were checked by co-authors who are HCPs themselves (DKW, LVB), and they agreed with the themes that emerged.
Reporting		
29. Quotations presented	Were participant quotations presented to	Yes, participant quotations were described and identified with a participant number.

S1 Appendix. COREQ checklist.

COREQ checklist: "Perceptions of cancer survivors and healthcare professionals on adequate supportive care after treatment of cancer: an in-depth qualitative study"

	illustrate the themes / findings? Was each quotation identified? e.g. participant number	
30. Data and findings consistent	Was there consistency between the data presented and the findings?	Yes.
31. Clarity of major themes	Were major themes clearly presented in the findings?	Yes, these are indicated in the results section of the paper.
32. Clarity of minor themes	Is there a description of diverse cases or discussion of minor themes?	Yes, these are indicated in the results section of the paper.
