# Supplementary material for: Perceptions of cancer survivors and healthcare professionals on adequate supportive care after treatment of cancer: An in-depth qualitative study
Source: PLoS One. 2026 Aug 25;21(8):e0355113. doi: 10.1371/journal.pone.0355113 (PMC13505917; doi:10.1371/journal.pone.0355113)
Supplement: Supplementary file 2 [file pone.0355113.s002.pdf]

S2 Appendix. Topic guide: interviews with cancer survivors.

<u>Experiences with and perspectives on supportive care and guidance</u>

1. What are your experiences with supportive care and guidance?
2. How did you experience communication and decision-making regarding supportive care and guidance?
3. Do you have any suggestions for improvement?
4. Are you currently in need of supportive care and guidance?
    a. If yes, what kind of supportive care and guidance?
    b. What should supportive care and guidance focus on?
5. Did you previously need supportive care and guidance?
    a. If yes, what kind of supportive care and guidance?
    b. What did the supportive care and guidance focus on?
    c. At what point in time did you need it?
6. At what point in the care pathway should supportive care and guidance be offered?
    a. Supportive care and guidance within the hospital, or outside the hospital? E.g. by healthcare professional (inside hospital) or coach, fellow sufferers contact (outside hospital)
7. Did your loved ones need supportive care and guidance?
8. From which healthcare professional would you like to receive supportive care and guidance?
    a. Psychologist, general practitioner, social worker, peers, etc.
9. What would you expect from your healthcare professional?
    a. What do you think a good conversation might look like?
10. What do you think is most important about supportive care and guidance?
