# Supplementary material for: Perceptions of cancer survivors and healthcare professionals on adequate supportive care after treatment of cancer: An in-depth qualitative study
Source: PLoS One. 2026 Aug 25;21(8):e0355113. doi: 10.1371/journal.pone.0355113 (PMC13505917; doi:10.1371/journal.pone.0355113)
Supplement: Supplementary file 3 [file pone.0355113.s003.pdf]

S3 Appendix. Topic guide: focus groups with healthcare professionals.

Experiences with and perspectives on supportive care and guidance

1. Experiences in providing supportive care and guidance to cancer survivors
   a. What types of supportive care and guidance do you currently provide?
   b. Who currently has what role in providing supportive care and guidance in your organization?
   c. What supportive care and guidance do you currently refer patients to?
   d. Based on what information do you currently offer supportive care and guidance?
   e. To what extent are supportive care and guidance currently tailored to individual needs of patients?
   f. What are your experiences with deciding on supportive care and guidance with the patient?
2. Perspective on appropriate supportive care and guidance
   a. To what extent does current supportive care and guidance match what patients want/need?
   b. How do you think supportive care and guidance could be improved? What is needed to achieve this?
   c. What do you think optimal/appropriate supportive care and guidance looks like?
      - For whom (patients or also next of kin)?
      - When offered in the medical pathway?
      - Focused on what?
      - Offered by whom? (e.g. professionals, fellow sufferers)
      - In what form?
   d. Which care healthcare professional ideally has which role?
   e. How do you collaborate with different healthcare professionals in the organization?
   f. As healthcare professionals, what do you need to better align supportive care and guidance with patients' individual needs?
   g. What do you need to make shared decisions about supportive care and guidance with patients?
