# Supplementary material for: Perceptions of cancer survivors and healthcare professionals on adequate supportive care after treatment of cancer: An in-depth qualitative study
Source: PLoS One. 2026 Aug 25;21(8):e0355113. doi: 10.1371/journal.pone.0355113 (PMC13505917; doi:10.1371/journal.pone.0355113)

S4 Appendix. Coding tree.

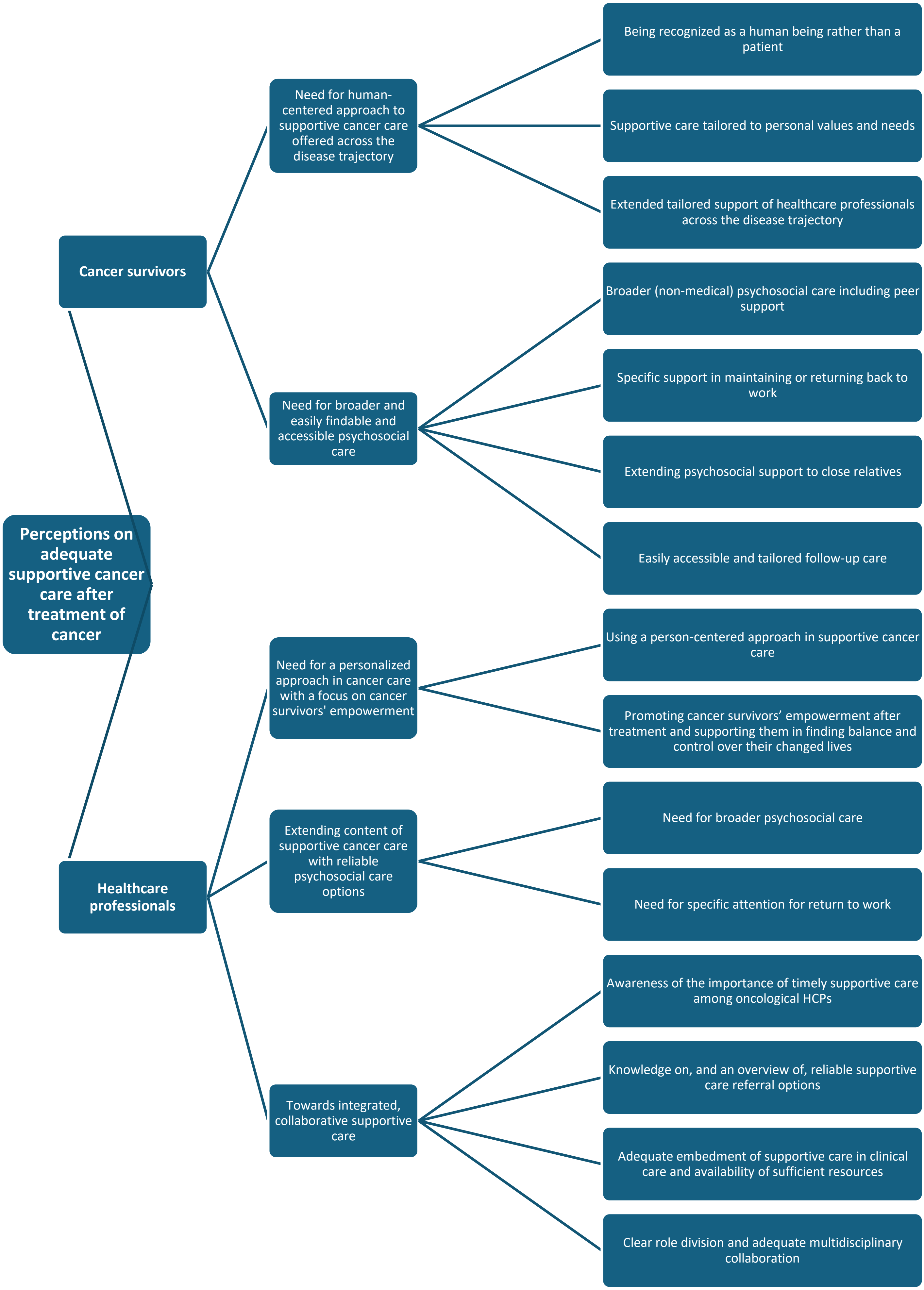

Supplement: Supplementary file 4 [file pone.0355113.s004.pdf]
